# Bringing HIV services to key populations and their communities in Tanzania: from pilot to scale

**DOI:** 10.1002/jia2.25718

**Published:** 2021-06-30

**Authors:** Haruka Maruyama, Julie Franks, Damian Laki, Omari Msumi, Neema Makyao, Oscar E Rwabiyago, Miriam Rabkin, Magreth J Kagashe, Wafaa M El‐Sadr

**Affiliations:** ^1^ ICAP at Columbia University Dar es Salaam Tanzania; ^2^ Mailman School of Public Health ICAP at Columbia University New York NY USA; ^3^ National AIDS Control Programme Ministry of Health, Community Development Gender, Elderly and Children (MoHCDGEC) Dodoma Tanzania; ^4^ Centers for Disease Control and Prevention in Tanzania Dar es Salaam Tanzania

**Keywords:** key and vulnerable populations, differentiated care, HIV care continuum, community, testing, linkage to care

## Abstract

**Introduction:**

Despite the global scale‐up of HIV testing, prevention and treatment, these services remain inaccessible to groups most vulnerable to HIV. Globally, most new HIV infections are concentrated among members of key populations (KP), including female sex workers, men who have sex with men, transgender people, people who inject drugs and their sexual partners. These populations lag in access to HIV prevention and antiretroviral therapy (ART) and have less favourable HIV outcomes compared to the general population. Intersecting behavioural and structural factors contribute to these gaps in service access for at‐risk KP and those living with HIV; corresponding comprehensive approaches to improving service delivery for KP are urgently needed. Differentiated service delivery (DSD) models tailor HIV programmes to the needs and preferences of specific groups but are rarely implemented at scale for KP. We describe the FIKIA Project, which implemented innovative approaches to scaling up DSD models to reach and engage KP in Tanzania.

**Methods:**

The FIKIA Project worked with diverse KP communities in Tanzania to tailor HIV services to their needs and to pair healthcare workers with trained peer educators and expert client counsellors to expand uptake of community‐based HIV testing and ART services. We analysed routine aggregate project data from 2016 to 2020 to describe project implementation, outcomes and best practices.

**Results and discussion:**

The FIKIA Project conducted 1,831,441 HIV tests in community settings; of the 98,349 (5.4%) individuals with new HIV diagnoses, 89,640 (91.1%) initiated ART. The project reached substantial numbers of KP: 203,233 received HIV tests, 28,830 (14.2%) received a new HIV diagnosis and 25,170 KP (87.3%) initiated ART at the point of diagnosis. Over time, HIV testing increased by 1.6 times overall (2.3 times among KP), HIV diagnoses increased by 8.7 times (10.9 times among KP) and ART initiation at the point of diagnosis increased from 80.0% to 95.9% overall (from 69.6% to 94.9% among KP).

**Conclusions:**

Over four years, the FIKIA Project scaled up HIV testing, diagnosis and treatment by using DSD principles to design services that meet the needs of KP and their communities.

## 1 INTRODUCTION

Despite the scale‐up of effective, evidence‐based interventions for HIV testing, prevention and treatment, key populations (KP), including female sex workers (FSW), men who have sex with men (MSM), transgender people and people who inject drugs (PWID), lack access to these critical services. Globally, KP and their sexual partners accounted for over 60% of new HIV infections among adults in 2019, highlighting the urgent need to reach them with HIV services [1]. KP living with HIV also have poorer access to antiretroviral therapy (ART) and are less likely to have sustained viral suppression than other groups [2]. These gaps result from intersecting socio‐behavioural and structural factors that contribute to high HIV rates and poor treatment outcomes.

Innovative approaches to service delivery have improved the accessibility, quality, acceptability and efficiency of HIV programmes [3, 4]. Differentiated service delivery (DSD) models enable health systems to tailor programmes to the needs and preferences of specific groups, while safeguarding the public health approach necessary to take programmes to scale in resource‐constrained settings [5]. By adjusting where HIV services are provided, who provides the services, how frequently services are offered and what services are included, DSD models offer person‐centred, contextually appropriate care for diverse groups of persons living with HIV (PLWH). Many countries are scaling up DSD and sharing lessons learned; for example the Coverage, Quality and Impact Network (CQUIN) [6], a learning network, enables 21 member countries in sub‐Saharan Africa, including Tanzania, to exchange relevant best practices and resources. DSD models for KP are typically small pilot projects rather than large‐scale programmes [7]. Barriers to KP‐focused DSD include lack of national mandates, policy barriers and the need for contextually tailored programme designs. Although pilot projects are important to identify and highlight best practices, they often occur in controlled settings that may not be replicable at scale. Reaching KP and providing sustained engagement and support for HIV services to the often economically and geographically marginalized communities where they reside is also essential to control the HIV epidemic [8, 9].

Tanzania has a generalized HIV epidemic with 4.8% prevalence among adults and approximately 1.7 million PLWH [10]. The 2016 to 2017 population‐based HIV impact assessment (PHIA) survey in Tanzania found that only 61% of adults living with HIV were aware of their status; of these, 94% were receiving ART; and of these, 87% had viral load suppression [11]. Tanzania also has a concentrated epidemic among KP groups, including FSW, MSM and PWID [12]. Consensus estimates from 2014 for mainland Tanzania indicate approximately 155,450 FSW with an estimated 26% HIV prevalence, approximately 49,700 urban MSM with 25% HIV prevalence and 30,000 PWID with 36% HIV prevalence [13]. Sex work, drug use and same‐sex sexual relationships are criminalized in Tanzania, complicating efforts to engage these populations in accordance with the 2017 National Guideline for Comprehensive Package of HIV Interventions for Key and Vulnerable Populations. Despite the important strides Tanzania has taken to expand community‐based HIV services to reach these populations, gaps in service delivery remain.

In response to these service delivery gaps, ICAP at Columbia University partnered with the Ministry of Health, Community Development, Gender, Elderly and Children (MoHCDGEC) and the US Centers for Disease Control and Prevention (CDC) to design and implement the FIKIA Project (Swahili for “to reach”) to take traditionally facility‐based HIV services into communities to broaden service reach, acceptability and uptake.

## 2 METHODS

### 2.1 Project time frame

FIKIA launched in October 2016 with five‐year funding from the US President’s Emergency Plan for AIDS Relief (PEPFAR) via the US Centers for Disease Control and Prevention. This analysis reports on data from October 2016 to September 2017 (Year 1), October 2017 to September 2018 (Year 2), October 2018 to September 2019 (Year 3) and October 2019 to September 2020 (Year 4).

### 2.2 Geographic coverage and project targets

Annual targets for HIV services including testing and yield of new HIV diagnoses from all tests performed overall and for specific populations are noted in Table 1, which shows the evolution of targets adapted to emerging national priorities. Geographic coverage of regions and districts changed annually, and included Dar es Salaam, Pwani, Tanga, Kagera, Mwanza, Kigoma, Mara, Simiyu and Geita. PEPFAR and MoHCDGEC priorities also led to adjustments in project targets with increased emphasis on FSW, who were expected to comprise 80% of KP‐specific service delivery targets in Years 3 and 4.

**Table 1:** ICAP’s FIKIA Project geographic coverage and project targets with corresponding results for community‐based HIV services in PEPFAR‐CDC supported regions in Tanzania, October 2016 to September 2020

Variable	Year 1	Year 2	Year 3	Year 4^a^
Geographic coverage^b^				
Total number of regions	9	9	9	8
Total number of districts	23	57	49	47
Targets^c^				
HIV testing – all population	361,323	522,785	819,652	280,911
HIV positive – all population	TNS	13,022	39,323	35,515
HIV yield – all population	TNS	2.5%	4.8%	12.6%
Reach with HIV services (all KP)	29,841	55,104	60,249	57,122
Reach with HIV services (FSW)	TNS	TNS	48,268	45,183
Reach with HIV services (MSM)	TNS	TNS	6552	6468
Reach with HIV services (PWID)	TNS	TNS	5429	5471
Results^d^				
HIV testing – all population	157,718	542,911	874,003	256,809
HIV positive – all population	4310	14,379	42,042	37,618
HIV yield – all population	2.7%	2.6%	4.8%	14.6%
Reach with HIV services (all KP)	23,028	63,664	70,340	69,267
Reach with HIV services (FSW)	17,146	52,265	56,398	56,065
Reach with HIV services (MSM)	1740	5103	6766	7105
Reach with HIV services (PWID)	4142	6296	7176	6097

FSW, female sex workers; KP, key populations; MSM, men who have sex with men; PWID, people who inject drugs; TNS, target not set (the target was not set by PEPFAR in that year).

^a^ Time period covers the following: Year 1 = October 2016 – September 2017; Year 2 = October 2017 – September 2018; Year 3 = October 2018 – September 2019; Year 4 = October 2019 – September 2020

^b^ Geographic coverage included regions, each with a varying number of districts that were covered by the project

^c^ HIV‐testing targets (HTS_TST in the PEPFAR MER – Monitoring Evaluation, and Reporting – Indicator Reference Guide) [14], HIV‐positive targets (HTS_POS in the PEPFAR MER Guide) and targets to reach KP groups with HIV services (KP_PREV in the PEPFAR MER Guide, where being “provided, offered or referred” HIV testing was a required component for the indicator) were set annually by the donor in consultation with MoHCDGEC

^d^ Corresponding programme results that were achieved against the target indicator are provided for each time period, regardless of if the target was set or not.

### 2.3 Project design

FIKIA’s approach to reaching KP was centred on fundamental principles of DSD: providing client‐centred HIV services in the most appropriate manner for specific groups. FIKIA teams travelled by motorcycle, mobile clinic, car, and/or boat to provide services wherever clients lived, worked and socialized: on small, off‐grid Lake Victoria islands; in informal gold mines; outside city markets; in remote rural villages; and inside brothels and drug‐use camps. Locations were also tailored to KP groups: for example “backpack testing” was used for MSM, in which a healthcare worker in tandem with a MSM peer worker arranged for testing one or several clients in a community location of the clients’ choice. To optimize access, FIKIA staff organized services at times convenient for clients, including evenings and weekends. The timing was also customized to KP group; for example HIV testing was scheduled in the evening in mobile units near workplaces for FSW, when they were more likely to be available.

The types of services delivered were tailored to clients’ needs. During community‐based activities, comprehensive, client‐centred biomedical services including HIV counselling and testing and immediate ART initiation were delivered by facility healthcare workers trained in national HIV testing, prevention and treatment guidelines and provision of KP‐friendly services by national facilitators from MoHCDGEC. All HIV tests were conducted according to the national testing algorithm, and eligibility for ART was in accordance with national guidance on test‐and‐start. Strategies to enhance targeted HIV testing included brief eligibility screening based on symptoms and risk, index testing and partner notification services following PEPFAR and World Health Organization guidance for safe and ethical index testing, and social network testing. Intervention approaches also were tailored to populations; for example HIV testing was integrated with preparatory counselling for medication‐assisted treatment (MAT) for PWID.

Lastly, close attention was paid to the “who” of DSD – the cadres delivering FIKIA services. All community‐based activities were supported by more than 1,000 trained peer educators and expert clients, including KP and PLWH who identified appropriate service locations and safe spaces, conducted screening to identify potential beneficiaries, organized peer education and counselling and facilitated navigation to biomedical services. Capacity‐building of KP‐led civil society organizations (CSO) was also an essential component of the FIKIA approach. KP CSOs collaborated with FIKIA teams to ensure services responded to beneficiary needs and preferences. Partnership and funding agreements were structured to reinforce clear shared expectations of outputs and outcomes, regular communication and identification of CSO capacity‐building needs. CSOs received support for staffing, supplies and operational costs to implement specific activities, such as providing psychosocial and adherence support to PWID clients receiving MAT and mentoring peer educators who were conducting outreach.

### 2.4 Data collection and analysis

Project data for community‐based HIV testing, HIV diagnosis and ART initiation were collected using paper‐based national MoHCDGEC tools. Data were entered into electronic databases, including ICAP’s customized District Health Information Software platform using PostgreSQL, and were aggregated for monthly and quarterly reporting to MoHCDGEC and PEPFAR and for reporting to local governmental and health authorities and at the national level through technical working groups and other stakeholder meetings. Descriptive analyses were performed to explore trends over time in the number of HIV tests conducted, number and proportion (yield) of HIV diagnoses made and number and proportion of people initiating ART. The results were disaggregated to compare the overall population with KP and were analysed over time by project year.

## 3 RESULTS AND DISCUSSION

### 3.1 Results

Of the 1,831,441 HIV tests conducted through FIKIA, 98,349 (5.4%) resulted in new HIV‐positive diagnoses, and 89,640 persons (91.1% of those with a new diagnosis) initiated ART. Of the total tests conducted, 203,233 (11.1%) were among KP, with 28,830 (14.2% yield among KP) HIV‐positive diagnoses and 25,170 KP members (87.3% of those with a new diagnosis) initiated on ART (Table 2). Overall HIV testing and HIV‐positive targets as well as KP‐specific targets were met and surpassed each year (Table 1), except for Year 1, a start‐up year in which service delivery was launched in the latter half of the year. Between Year 3 and Year 4, testing numbers substantially decreased from 874,003 to 256,809 consistent with a shift by PEPFAR to focus on targeted testing and yield rather than the absolute number of tests.

**Table 2:** ICAP’s FIKIA Project results by year and population for community‐based HIV testing and ART initiation in PEPFAR‐CDC supported regions in Tanzania, October 2016–September 2020^a^

Variable	Time period	# HIV tests	# HIV positive	# ART initiated	% Yield	% ART initiated
All Population (with KP)	Year 1	157,718	4310	3448	2.7	80.0
	Year 2	542,911	14,379	11,935	2.6	83.0
	Year 3	874,003	42,042	38,195	4.8	90.8
	Year 4	256,809	37,618	36,062	14.6	95.9
	Total (year 1–4)^b^	1,831,441	98,349	89,640	5.4	91.1
KP Only (FSW + MSM + PWID)	Year 1	21,059	1052	732	5.0	69.6
	Year 2	63,148	5096	3502	8.1	68.7
	Year 3	69,669	11,249	10,090	16.1	89.7
	Year 4	49,357	11,433	10,846	23.2	94.9
	Total (year 1–4)	203,233	28,830	25,170	14.2	87.3
FSW only	Year 1	15,169	913	670	6.0	73.4
	Year 2	51,779	3855	2707	7.4	70.2
	Year 3	56,087	9163	8249	16.3	90.0
	Year 4	38,548	9251	8834	24.0	95.5
	Total (year 1–4)	161,583	23,182	20,460	14.3	88.3
MSM only	Year 1	1745	52	39	3.0	75.0
	Year 2	5081	498	308	9.8	61.8
	Year 3	6471	1055	960	16.3	91.0
	Year 4	5165	1123	1085	21.7	96.6
	Total (YEAR 1‐ 4)	18,462	2728	2392	14.8	87.7
PWID only	Year 1	4145	87	23	2.1	26.4
	Year 2	6288	743	487	11.8	65.5
	Year 3	7111	1031	881	14.5	85.5
	Year 4	5644	1059	927	18.8	87.5
	Total (year 1–4)	23,188	2920	2318	12.6	79.4

ART, antiretroviral therapy; FSW, female sex workers; KP, key population; MSM, men who have sex with men; PWID, people who inject drugs.

^a^ This table shows the FIKIA Project performance of community‐based HIV testing, diagnosis and ART initiation, along with % yield (#HIV positive/#HIV tests), and % ART initiation (#ART initiated/#HIV positive). All population covers the entire project performance, and subsets are presented for KP, including FSW, MSM and PWID

^b^ Time period covers the following: Year 1 = October 2016 – September 2017; Year 2 = October 2017 – September 2018; Year 3 = October 2018 – September 2019; Year 4 = October 2019 – September 2020.

**Figure 1:**
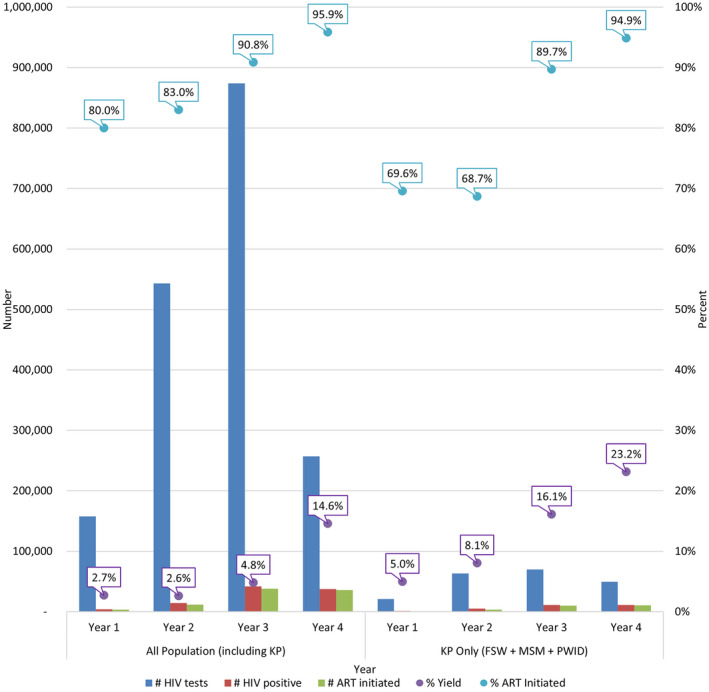
ICAP’s PEPFAR‐CDC supported FIKIA Project performance over time by total population and key population (KP) in Tanzania for community‐based testing and ART initiation, October 2016–September 2020. The bars (left y‐axis) indicate the number of HIV tests, HIV‐positive results and individuals who initiated antiretroviral therapy (ART). The dots (right y‐axis) indicate % yield (#HIV positive/#HIV tests), and % of individuals who initiated ART (#ART initiated/#HIV positive). The graph is divided into two parts – on the left, annual performance including all populations, and on the right, annual performance covering KP (including female sex workers (FSW), men who have sex with men (MSM) and people who inject drugs (PWID)). Time period covers the following: Year 1 = October 2016 – September 2017; Year 2 = October 2017 – September 2018; Year 3 = October 2018 – September 2019; Year 4 = October 2019 – September 2020

Over the four project years, the number of HIV tests, HIV‐testing yield (number of new HIV diagnoses) and ART initiation increased overall and among KP (Figure 1). The number of tests increased from 157,718 HIV tests in Year 1 to 256,809 HIV tests in Year 4, an increase of 1.6 times; of these, 21,059 tests in Year 1 and 49,357 tests in Year 4 were among KP, a 2.3 times increase. In the same time frame, overall number of HIV‐positive diagnoses increased 8.7 times (from 4,310 to 37,618) and HIV‐positive diagnoses among KP increased 10.9 times (from 1,052 to 11,433). This represented an increase over time in yield of HIV diagnoses from 2.7% to 14.6% among all populations and from 5.0% to 23.2% among KP.

With the rapid expansion of community‐based interventions, ART initiation markedly increased, both overall and for KP. In Year 1, 80.0% (3,448) of all individuals with a new HIV diagnosis initiated ART, compared to 95.9% (36,062) in Year 4. Among KP, 69.6% (732) of individuals with a new HIV diagnosis initiated ART in Year 1, compared to 94.9% (10,846) in Year 4. Similar trends over time were observed with increasing HIV identification and ART initiation rates among FSW, MSM and PWID (Table 2).

### 3.2 Discussion

Prior to the FIKIA Project, there was neither a coordinated national KP initiative nor a dedicated PEPFAR‐CDC funded community‐based implementing partner in the operating regions. KP services were not formalized or available at scale in health facilities (apart from MAT clinics in Dar es Salaam) or communities. Preceding this project, wide‐scale HIV testing and ART services in the community were not available in the targeted regions.

The FIKIA Project used DSD principles to rapidly scale up community‐based HIV testing and ART initiation, including among KP residing within these communities. The location of services was critical, not only to relieve clients of the time and cost of travel to distant facilities but also to increase service delivery without placing additional burdens on health facilities. By bringing services into communities, FIKIA was able to tailor who provided these services. KP peers and expert clients were recruited and employed in local areas, where they were well‐positioned to identify optimal locations and times to engage clients and to educate community members on the benefits of services. FIKIA’s mobile approach to service delivery facilitated adjustments to how frequently services would be provided, such as backpack HIV testing to reach MSM, partners of newly diagnosed PLWH and members of PWID social networks. Equally important, FIKIA tailored which services were offered, providing ART initiation at the point of diagnosis in the community and MAT preparation along with HIV services for PWID. FIKIA’s use of the DSD approach enabled reaching KP as well as other at‐risk populations in their communities.

The success of the project can be attributed to the close partnership between ICAP teams and the MoHCDGEC as well as regional‐level and district‐level governmental units. This partnership facilitated the rapid adoption of KP and DSD services into national policies and guidelines and motivated the implementation of systems for procurement, training and monitoring and evaluation. The project demonstrated the importance of working with government structures in the design and implementation of services, and frequent sharing of programmatic data‐informed decision making at regional and national levels and facilitated the adoption and scale‐up of programme innovations. On the basis of results from FIKIA, the National AIDS Control Programme at the MoHCDGEC added outreach HIV testing and community ART initiation to the 2019 National HIV Treatment Guidelines.

Our findings are subject to several limitations. There are inherent challenges in using programmatic aggregate data that limit the ability to conduct individual‐level analyses. As service delivery targets were determined by the funder for specific groups in defined geographic districts, it was not possible to determine overall coverage with these services for specific populations or geographic regions.

## 4 CONCLUSIONS

The FIKIA Project in Tanzania successfully scaled up HIV services to reach critical populations by adapting DSD principles to meet the needs of KP and their communities. Expanding access to client‐centred services is critical to reaching and engaging KP who need HIV services. We can learn from the “science of scale‐up” and create innovative strategies to leverage the lessons of KP pilot programmes and take them to scale through adopting DSD principles and attention to community and client needs and preferences. Community engagement approaches and client‐centred service delivery methods used during FIKIA implementation in Tanzania hold important lessons for optimizing scale‐up of targeted HIV services for KP and other at‐risk populations in other settings.

## Competing interests

The authors have declared no conflict of interest.

## Authors’ contributions

H.M. and J.F. designed and directed the project and developed the main conceptual ideas and writing of the manuscript in close consultation and review with M.R. and W.E.S. D.L. oversaw data collection, provided numerical results for the manuscript and verified the figures and tables. O.M was involved in planning and supervising the project as well as developing the methods and background sections. O.E.R, N.M and M.J.K. guided the project planning, implementation and scale‐up as part of the funder and government agencies, respectively, and participated in critical review and approval of the manuscript content. All authors met authorship criteria as defined by the ICMJE and Journal of the International AIDS Society.

## Abbreviations

ART, antiretroviral therapy; CSO, civil society organization; DSD, differentiated service delivery; FSW, female sex workers; KP, key populations; MAT, medication‐assisted treatment; MoHCDGEC, Ministry of Health, Community Development, Gender, Elderly and Children; MSM, men who have sex with men; PEPFAR, President’s Emergency Plan for AIDS Relief; PLWH, people living with HIV; PWID, people who inject drugs.
